# Spatial Transcriptomics Reveals Distinct Architectures but Shared Vulnerabilities in Primary and Metastatic Liver Tumors

**DOI:** 10.3390/cancers17193210

**Published:** 2025-10-01

**Authors:** Swamy R. Adapa, Sahanama Porshe, Divya Priyanka Talada, Timothy M. Nywening, Mattew L. Anderson, Timothy I. Shaw, Rays H. Y. Jiang

**Affiliations:** 1 USF Genomics Program, College of Public Health, University of South Florida, Tampa, FL 33612, USA; 2 Department of Global, Environmental, and Genomic Health Sciences, College of Public Health, University of South Florida, Tampa, FL 33612, USA; 3 Morsani College of Medicine, University of South Florida, Tampa, FL 33612, USA; 4 Department of Biostatistics and Bioinformatics, H. Lee Moffitt Cancer Center & Research Institute, Tampa, FL 33612, USA; 5 Department of Surgery, University of South Florida, 560 Channelside, Drive, Tampa, FL 33602, USA; 6 Department of Obstetrics and Gynecology, Morsani College of Medicine, University of South Florida, Tampa, FL 33606, USA

**Keywords:** tumor microenvironment, plasticity, porphyrin metabolism, heme metabolism, prostaglandin, cancer metabolism

## Abstract

**Background**: Primary hepatocellular carcinoma (HCC) and liver metastases differ in origin, progression, and therapeutic response, yet a direct high-resolution spatial comparison of their tumor microenvironments (TMEs) within the liver has not previously been performed. **Methods**: We applied high-definition spatial transcriptomics to fresh-frozen specimens of one HCC and one liver metastasis (>16,000 genes per sample, >97% mapping rates) as a proof-of-principle two-specimen study, cross-validated in human proteomics and patients’ survival datasets. Transcriptional clustering revealed spatially distinct compartments, rare cell states, and pathway alterations, which were further compared against an independent systemic dataset. **Results**: HCC displayed an ordered lineage architecture, with transformed hepatocyte-like tumor cells broadly dispersed across the tissue and more differentiated hepatocyte-derived cells restricted to localized zones. By contrast, liver metastases showed two sharply compartmentalized domains: an invasion zone, where proliferative stem-like tumor cells occupied TAM-rich boundaries adjacent to hypoxia-adapted tumor-core cells, and a plasticity zone, which formed a heterogeneous niche of cancer–testis antigen–positive germline-like cells. Across both tumor types, we detected a conserved metabolic program of “porphyrin overdrive,” defined by reduced cytochrome P450 expression, enhanced oxidative phosphorylation gene expression, and upregulation of *FLVCR1* and *ALOX5*, reflecting coordinated rewiring of heme and lipid metabolism. **Conclusions**: In this pilot study, HCC and liver metastases demonstrated fundamentally different spatial architectures, with metastases uniquely harboring a germline/neural-like plasticity hub. Despite these organizational contrasts, both tumor types converged on a shared program of metabolic rewiring, highlighting potential therapeutic targets that link local tumor niches to systemic host–tumor interactions.

## Simple Summary

Spatial transcriptomics reveals that primary and metastatic liver tumors have distinct architectures but share a conserved metabolic vulnerability.

## 1. Introduction

Primary hepatocellular carcinoma (HCC) and liver metastases are among the most common malignant tumors affecting the liver, yet they differ fundamentally in origin, pathogenesis, and therapeutic response [1,2]. HCC arises from hepatocytes, often in the context of chronic liver disease, and its growth and progression are influenced by hepatic zonation, vascular structure, and the underlying inflammatory milieu [3,4]. Liver metastases, by contrast, arise from non-hepatic primaries, commonly colorectal cancer, where colonic epithelial cells disseminate hematogenously through the portal vein and adapt to the hepatic microenvironment [5]. Despite these distinct trajectories, both tumor types share the challenge of remodeling liver tissue to support growth, evade immune surveillance, and exploit local metabolic resources.

The tumor microenvironment (TME) plays a central role in these processes, integrating immune, stromal, and vascular components into a dynamic ecosystem that shapes tumor behavior [5]. Immune–stromal aggregates such as tertiary lymphoid structures have been recognized as important sites of local immunity in solid tumors [6,7], but their composition, stromal scaffolding, and functional roles vary widely between tumor types. Likewise, tumor cell plasticity, including partial epithelial–mesenchymal transition (pEMT), lineage dedifferentiation, and the emergence of germline- or neural-like phenotypes, has been linked to invasion, therapy resistance, and metastatic progression [8,9,10]. Understanding how these features are spatially organized within the tumor landscape is essential to identifying actionable vulnerabilities.

Metabolic reprogramming is a hallmark of cancer, enabling cells to sustain proliferation, survive metabolic stress, and adapt to microenvironmental constraints [11,12,13]. In the liver, pathways linked to heme and porphyrin metabolism are of particular interest [14], as this organ is one of the principal sites of heme biosynthesis, degradation, and trafficking [15,16,17]. Heme is essential for oxygen transport, mitochondrial respiration, and redox regulation [15], while its biosynthetic intermediates, such as protoporphyrin IX (PPIX), can influence oxidative stress and cell fate [18,19,20]. Alterations in enzymes of the heme biosynthesis pathway, such as ALAS1, HMBS, and FECH, or in export transporters like FLVCR1 can disrupt the balance between production and clearance, leading to accumulation of porphyrin intermediates [13,18,20]. Such imbalances may affect mitochondrial function, ferroptosis sensitivity, and immune modulation. Recent evidence suggests that both primary and metastatic liver tumors can develop a “porphyrin overdrive” phenotype [14,21], with reduced mature heme synthesis, intermediates (porphyrin) accumulation, and upregulation of porphyrin export and electron transport chain activity. These metabolic shifts may be further intertwined with inflammatory lipid pathways [12,22,23,24], including ALOX5-mediated prostaglandin signaling, which has been implicated in tumor–stroma–immune crosstalk [22,24,25].

Spatial transcriptomics offers a powerful means to interrogate these biological processes in situ, preserving spatial context and enabling the mapping of tumor, stromal, and immune populations alongside their molecular programs [26,27,28,29]. By integrating transcriptional and histological data, spatial profiling can reveal restricted cell states, microenvironmental niches, and metabolic adaptations that are not apparent from dissociated single-cell analyses [4,24,26]. The liver represents a particularly distinctive context, shaped by metabolic zonation, dual blood supply, and an immune-tolerant microenvironment, all of which condition tumor behavior. While large-scale atlases [26,30] have provided broad cross-cancer insights, a focused spatial comparison of primary HCC and liver metastases within the hepatic microenvironment has not yet been undertaken.

In this study, we applied high-definition spatial transcriptomics to human HCC and liver metastasis in a two-specimen study to systematically compare their spatial architectures, cellular composition, immune organization, and metabolic reprogramming. We also reanalyzed an independent dataset [31] from tumor-bearing mice to investigate whether tumor-driven metabolic changes extend to uninvolved liver tissue. Our goal was to define tumor-specific features and conserved vulnerabilities, particularly in heme–porphyrin metabolism and immune–stromal organization, that could inform precision therapeutic strategies for liver cancer.

## 2. Materials and Methods

### 2.1. Human Samples

De-identified human liver tissues were obtained from BioIVT (commercial biobank, Westbury, NY 11590, USA) under institutional approval for use of commercially sourced, de-identified specimens. Two treatment-naïve cases were analyzed: (i) primary hepatocellular carcinoma (HCC) from a 70-year-old Caucasian male, moderately differentiated; and (ii) liver metastasis from a 71-year-old male, segmentectomy specimen with a 9 cm metastatic adenocarcinoma. Tissues were provided fresh-frozen and processed for high-definition spatial transcriptomics as described below. No patient-identifying information was available to the investigators.

### 2.2. Visium HD Spatial Transcriptomics

High-definition spatial transcriptomics was performed using the 10x Genomics Visium CytAssist HD platform according to the manufacturer’s protocol. Fresh-frozen 8 µm sections from primary HCC and liver metastasis were mounted on Visium HD slides, fixed, H&E-stained, imaged, and processed for cDNA synthesis and library construction. Sequencing was performed on an Illumina platform, and data were processed using Space Ranger v3.0 (10x Genomics) with alignment to the human reference genome and probe set. For both specimens, default Visium HD binning (8 µm bins, underlying 2 µm features) was applied. To ensure quality, bins were retained if they contained ≥200 UMIs, ≥150 detected genes, and ≤20% mitochondrial transcripts.

For the HCC specimen, a total of 372,936 binned 8 µm squares under tissue were captured, representing 53.1% of the slide area. Sequencing generated 898,165,296 reads, with a mean of 2408.4 reads and 541.5 UMIs per bin. A total of 16,655 genes were detected, corresponding to 8,461,395 UMIs and 34,014,388 reads per mm^2^ of tissue. Mapping quality was high, with 97.9% of reads aligning to the probe set, 96.9% mapping confidently, and 94.6% mapping to the filtered probe set. Valid barcodes accounted for 92.6% of reads, UMIs were 100% valid, and sequencing saturation was 74.0%. Q30 base percentages were 96.1% for barcodes, 95.7% for probe reads, and 96.7% for UMIs.

For the liver metastasis specimen, 418,456 binned 8 µm squares under tissue were captured, representing 59.6% of the slide area. Sequencing produced 733,847,760 reads, with a mean of 1753.7 reads and 218.1 UMIs per bin. In total, 16,868 genes were detected, corresponding to 3,408,103 UMIs and 24,839,443 reads per mm^2^. Mapping rates were similarly high, with 98.4% of reads aligning to the probe set, 97.5% mapping confidently, and 95.0% mapping to the filtered probe set. Valid barcodes accounted for 94.8% of reads, UMIs were 100% valid, and sequencing saturation reached 85.7%. Q30 base percentages were 96.3% for barcodes, 95.7% for probe reads, and 96.8% for UMIs.

### 2.3. Data Processing, Clustering, and Analysis

Sequencing data were processed using Space Ranger (10x Genomics, v3) with default parameters and the Visium HD human probe set. Only bins located under tissue with valid barcodes and unique molecular identifiers (UMIs), as determined by Space Ranger, were retained for downstream analysis. Gene expression matrices were imported into R (v4.2.2) for normalization, scaling, and dimensionality reduction, with low-quality bins excluded according to 10x Genomics’ default thresholds.

Differentially expressed genes (DEGs) were computed on normalized expression matrices using the pipeline’s default non-parametric framework. Pairwise contrasts were performed between (i) unsupervised clusters and (ii) user-defined spatial zones (e.g., Zone Invasion vs. Zone Plasticity) or selected cell groups (e.g., Hep-like tumor vs. Hep-pericentral). Genes were tested using the Wilcoxon rank-sum test, and *p*-values were adjusted with the Benjamini–Hochberg false discovery rate (FDR) correction. Unless otherwise specified, DEGs were defined as those with FDR < 0.05, a minimum expression fraction of 10% in at least one group, and an absolute log_2_ fold-change > 0.25. Marker lists for each cluster were generated by one-vs-rest comparisons under the same thresholds. Genes with near-zero counts in >90% of bins were excluded prior to testing to minimize spurious hits.

Unsupervised clustering was performed using principal component analysis (PCA) followed by shared nearest neighbor (SNN) modularity optimization. Low-dimensional visualizations were generated with Uniform Manifold Approximation and Projection (UMAP). Spatial distributions of clusters were visualized in Loupe Browser (10x Genomics) using coordinates generated by Space Ranger.

To quantitatively assess spatial structure, Moran’s I statistics [32] were computed in R (v4.3.2) using the spdep package (v1.3-4). For each cluster, bin-level coordinates from the Visium HD output were combined with cluster assignments, and spatial neighbor graphs were constructed using k-nearest neighbors (k = 4). Binary vectors indicating cluster membership were tested for spatial autocorrelation against randomly permuted backgrounds. Reported values include Moran’s I statistic, standardized z-scores, and *p*-values based on permutation tests.

Cell group annotation was based on DEGs with log_2_ fold-change > 0 and adjusted *p* < 0.05, supported by gene set enrichment analysis (GSEA v4.4) to confirm pathway-level concordance. Identities were assigned through manual curation of transcriptional signatures, integrating spatial localization, canonical lineage markers, and functional enrichment. Pathway enrichment analysis of DEGs was performed using Kyoto Encyclopedia of Genes and Genomes (KEGG) databases. Heme and porphyrin metabolism gene sets were compiled from KEGG v115 pathway hsa00860 and literature sources, while electron transport chain (ETC) complex genes were evaluated separately to assess mitochondrial oxidative phosphorylation activity.

For the murine data analysis, publicly available single-cell RNA-seq and Visium spatial transcriptomic datasets from tumor-bearing and control mice (DDBJ accession numbers DRA014802 and DRA009332) were re-analyzed. Differential expression testing was performed between tumor-bearing and control clusters using Fisher’s exact test with Benjamini–Hochberg correction. Genes with an adjusted *p* < 0.05 and fold change > 2 were considered significant.

## 3. Results

High-definition spatial transcriptomics generated high-quality datasets for both HCC and liver metastases in this study, with >16,000 genes detected per specimen, mapping rates exceeding 97%, and Q30 scores above 95%. Tissue coverage spanned ~53% of the slide area for HCC and ~60% for liver metastasis, producing hundreds of millions of reads with sufficient depth for robust spatial gene expression profiling. The high resolution enabled detection of rare cellular states and precise mapping of fine anatomical boundaries, including TAM subgroup segregation, localized Hep-pericentral zones in HCC, and hepatocyte entrapment in metastatic invasions.

### 3.1. Dispersion Associated with Higher Transformation in Primary Liver Cancer

**Figure 1:**
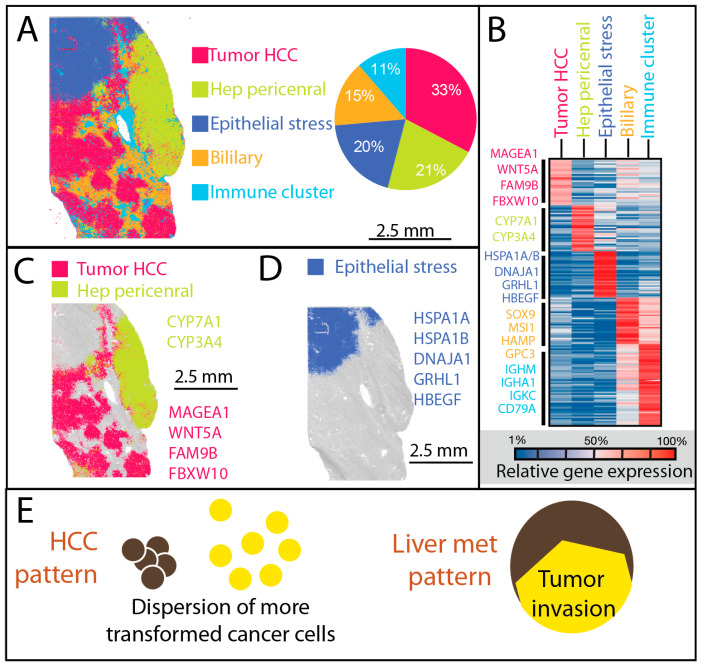
HCC Visium HD data with tissue overlay at single-cell resolution. (**A**) Cell types are color-coded according to the legend and shown with matching percentage distributions in the accompanying pie chart. The largest group, comprising approximately one-third of all cells, consists of tumor hepatocytes; (**B**) Heatmap of the top 50 marker genes for each cell group, with normalized expression values used as the scale; (**C**) Tumor hepatocytes display a dispersed, clump-like spatial pattern, whereas more differentiated hepatocellular carcinoma cells, likely derived from pericentral hepatocytes, are localized to a single anatomical region; (**D**) A cluster of epithelial cells of non-hepatocyte origin is positioned adjacent to tumor hepatocytes and exhibits elevated expression of stress-response genes, including multiple heat shock proteins. (**E**) Schematic summarizing the core spatial patterns observed in HCC versus liver metastasis. Primary HCC displays more differentiated tumor cells that cluster together and remain localized within defined regions, while transformed populations are more dispersed. The liver metastasis exhibits an epithelial-derived invasion zone, reflecting foreign lineage infiltration.

High-definition spatial transcriptomics of the HCC specimen revealed five major transcriptional compartments (Supplemental Tables S1 and S2), each with distinct molecular profiles and microanatomical localizations (Figure 1A–B). Supplemental Table S1 lists the top differentially expressed marker genes that were used to assign biological identities to each cluster (e.g., hepatocyte). Supplemental Table S2 summarizes the final cluster annotations. Hep-like tumor cells (33%) formed the dominant compartment, showing hepatocyte identity with stress-response and partial progenitor features. Hep pericentral cells (21%) retained hepatocyte zonation signatures and were often positioned near vascular structures. Epi stress cells (20%) represented a stress-adapted epithelial state enriched for immediate early gene activity, concentrated along invasive fronts and stromal interfaces. Biliary-like cells (15%) exhibited fetal-like inflammatory epithelial programs and localized to tumor peripheries and fibrotic septa. Immune stromal niches (11%) contained immune–stromal aggregates rich in plasma cells and fibroblasts at the capsule or fibrotic borders, with immune clusters embedded within these niches, displaying mature memory/follicular B-cell phenotypes and antigen-presentation capacity. In HCC, Moran’s I analysis confirmed non-random spatial organization across major clusters. Hep pericentral cells and epi stress cells showed the strongest autocorrelation (I = 0.81–0.92, *p* < 0.001), consistent with compact block-like architectures. In contrast, Hep-like tumor cells, biliary-like cells, and immune stromal niches displayed lower but significant autocorrelation (I = 0.59–0.72, *p* < 0.001), reflecting distribution in multiple discrete foci.

Two hepatocyte-derived malignant populations were distinguished by their degree of transformation and spatial organization. The more differentiated Hep pericentral cells, comprising about one-fifth of all cells, were confined to a single anatomical region, consistent with a localized growth pattern, while the more transformed Hep-like tumor cells, comprising one-third of all cells, were widely dispersed in clump-like foci across the tissue (Figure 1C). This broader dispersion correlated with higher transformation and suggests a capacity for more invasive or multifocal spread within the liver parenchyma.

At the tumor margin, a discrete population of non-hepatocyte epithelial cells expressing GRHL1 [33] was found in close proximity to hepatocyte-derived tumor cells (Figure 1D). These cells expressed elevated levels of stress-response genes, including multiple heat shock proteins, indicating adaptation to localized metabolic or immune pressures. Their juxtaposition with hepatocyte-derived cells highlights a spatial interface where lineage plasticity and stress adaptation may interact to shape the HCC microenvironment.

### 3.2. Spatial Complexity in Liver Metastasis

**Figure 2:**
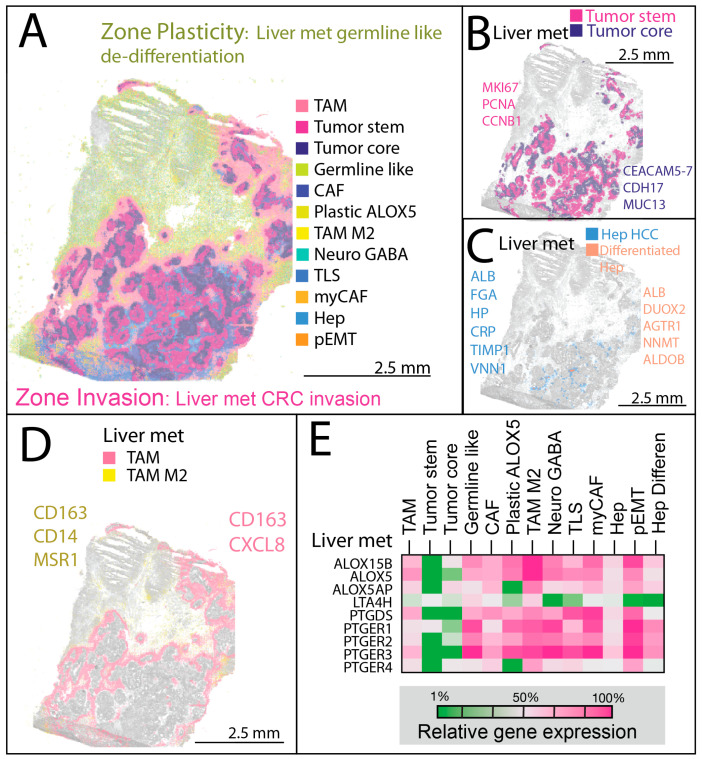
Liver metastasis Visium HD spatial transcriptomic data overlaid with tissue morphology. (**A**) Two distinct spatial zones are identified within the tumor. Zone Invasion represents the liver metastasis invasive front, where colorectal cancer cells are bordered by tumor-associated macrophages (TAMs) along the tumor edge. Zone Plasticity in metastasis contains germline-like, dedifferentiated cells composed of multiple highly plastic cell types, each shown in a different color; (**B**) The tumor compartment comprises rapidly proliferating, stem-like cells at the tumor periphery and a more differentiated tumor core with necrotic features; (**C**) Residual hepatocytes within the tumor invasion zone are interspersed among invading tumor cells, whereas more differentiated hepatocytes form small clumps resembling mature hepatocytes; (**D**) Two TAM subtypes are distinguished: one at the Zone Invasion boundary, forming a fringe around invading tumor cells and marking the interface between invading colonic epithelial cells and surrounding tissue; and an M2-like TAM population in Zone Plasticity, intermingled with germline-like and highly plastic cells; (**E**) Prostaglandin signaling in the liver metastasis. Expression of the inflammatory lipid-producing enzyme encoding gene *ALOX5* originates primarily from stromal rather than tumor epithelial cells. Both M2-like TAMs and plastic TAMs in Zone Plasticity, as well as pEMT cells, exhibit elevated *ALOX5* expression.

Spatial transcriptomic profiling of liver metastasis revealed a markedly more intricate architecture than HCC, with two sharply defined zones. A simplified schematic is provided in Figure 1E to illustrate the overall architectural differences observed between HCC and liver metastasis. Zone Invasion corresponded to the invasive front, where proliferative colorectal cancer cells were bordered by a dense fringe of tumor-associated macrophages (TAMs), forming a cellular barricade along the tumor edge (Figure 2A). Zone Plasticity represented a germline-like dedifferentiation domain containing multiple transcriptionally distinct, highly plastic cell types.

#### 3.2.1. Zone Invasion—Invasive Front and Structured Tumor–Stroma Interface

Zone Invasion was anchored by TAM (22.70%), a wound-repair fibrotic myeloid population forming a macrophage/stromal hybrid boundary. This compartment contained abundant tumor-stem (19.37%) cells, highly proliferative and stem-like with strong metabolic adaptability, and tumor-core (14.71%), a hypoxia-adapted, metabolically reprogrammed population localizing to perinecrotic regions (Supplemental Tables S3 and S4). These malignant compartments were assigned to colorectal-type origin based on strong expression of canonical intestinal lineage determinants [34,35,36,37] (e.g., *CDX2*, *CEACAM5*, *EPCAM*, *KRT20*) together with absence or very low of lineage-defining signatures from alternative primary sites, including lung [38] (*NKX2-1*), pancreas [39,40] (*PDX1*, *SOX9*), and breast [41,42] (*GATA3*, *FOXA1*). While colorectal carcinoma is the most parsimonious assignment, it is important to note that other gastrointestinal primaries, including appendix or small intestinal adenocarcinomas, can occasionally display overlapping immunoprofiles and may rarely express similar marker combinations [43,44]. Therefore, our assignment should be interpreted as most likely, though not absolutely exclusive. CAF (7.26%) contributed to extracellular matrix remodeling, while Hep (2.24%) and Hep-differentiated (0.22%) reflected residual hepatocytes. The immune–stromal aggregates (3.04%) spanned both zones but were often integrated with Zone Invasion’s fibroblastic networks.

Within Zone Invasion, rapidly proliferating, stem-like tumor cells concentrated at the periphery adjacent to TAM, while more differentiated Tumor-core cells with necrotic features occupied central regions (Figure 2B). Residual hepatocytes were interspersed within the invasion zone, harboring isolated clusters of mature hepatocyte-like cells resembling normal liver parenchyma (Figure 2C).

#### 3.2.2. Zone Plasticity—Dedifferentiation, Plasticity, and Immune Modulation

Zone Plasticity harbored transcriptionally diverse and plastic tumor states in liver metastasis in a confined physical location. Germline-like cells (9.20%) exhibited cancer-testis antigen and developmental signatures, suggesting metastatic stem-like potential. Plastic-ALOX5 (6.64%) represented an inflammatory, pluripotent tumor subset enriched for lipid-metabolic genes. TAM-M2 (4.16%) populated this zone with immunoregulatory and pro-angiogenic features, often co-localized with plastic tumor states. Stromal diversity included Neuro-GABA (4.14%) clusters with gene expression linked to neural mimicry and stress-adaptive signaling, myCAF (2.98%) with features of ECM-contractile activity. Rare pEMT-like cells (1.30%) at the invasive margin expressed both epithelial and mesenchymal programs. In the liver metastasis, Moran’s I analysis revealed distinct spatial modes across compartments. Tumor-stem and Tumor-core populations forming the invading tumor regions showed high spatial autocorrelation (I = 0.74–0.75, *p* < 0.001), consistent with compact invasion fronts. TAM-rich zones exhibited significant clustering in a zig-zag boundary pattern (I = 0.66, *p* < 0.001). Other lineages were more dispersed: germline-like cells and pEMT-like states showed intermediate autocorrelation (I = 0.43–0.62, *p* < 0.001). Together, these results quantitatively support the presence of both continuous invasion zones and multifocal, lineage-specific niches within the metastatic microenvironment.

TAM subtypes showed distinct spatial organization and functional polarization (Figure 2D). In Zone Invasion, TAMs (expressing *CD163* and *CXCL8*) formed a compact boundary layer at the tumor–stroma interface, displaying a fibro-inflammatory, angiogenic phenotype characterized by extracellular matrix remodeling, neovascular support, and residual inflammatory activity. In Zone Plasticity, TAM-M2 cells (expressing *CD163* and *MSR1*) were dispersed among germline-like and highly plastic tumor populations, adopting an immunosuppressive, tissue-repair-oriented program.

Prostaglandin pathway gene upregulation, reflecting inflammatory lipid metabolism, was concentrated in stromal niches, particularly within TAM and TAM-M2 populations, rather than in the bulk liver metastasis epithelium (Figure 2E) themselves. This pattern highlights lipid-mediated inflammatory signaling as a defining feature of the dedifferentiated microenvironment, consistent with previous reports by Soundararajan et al. [24].

### 3.3. Imbalanced Linear Heme Biosynthesis Pathway Genes Reveal “Porphyrin Overdrive” in Both Tumors

We previously described a dysregulated heme metabolic program in liver cancer, termed *porphyrin overdrive*, characterized by porphyrin accumulation and metabolic reprioritization [13,14]. In this model, liver cancer cells divert heme metabolism away from cytochrome P450-mediated detoxification and toward the electron transport chain (ETC), thereby fueling tumor bioenergetics and growth. This shift is marked by downregulation of hemoprotein genes in the P450 family and upregulation of ETC-associated genes during malignant transformation.

**Figure 3:**
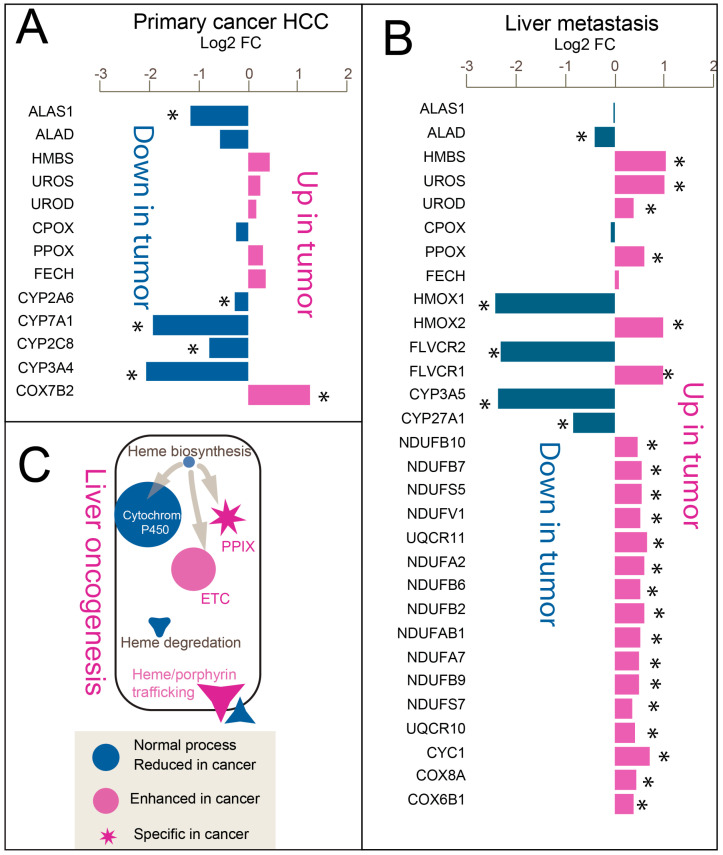
Heme and porphyrin metabolism gene expression in HCC and liver metastasis. Log_2_ fold changes are shown for comparisons between more aggressive and more differentiated tumor cell populations. * indicate significant differential expression with adjusted *p* value < 0.05. (**A**) HCC: Comparison of more transformed versus more differentiated hepatocyte-derived tumor cells. *ALAS1*, *ALAD*, *HMBS*, *UROS*, *UROD*, *CPOX*, *PPOX*, and *FECH* are heme biosynthesis genes. Cytochrome P450 enzymes are indicated by *CYP* genes, and electron transport chain (ETC) genes are denoted by *COX* genes; (**B**) Liver metastasis: Comparison of tumor stem-like outer zone cells versus tumor core cells. *HMOX1* and *HMOX2* are heme degradation genes; *FLVCR1* and *FLVCR2* encode heme/porphyrin exporters. ETC-related genes are marked, including Complex I (*NDUF* genes), Complex III (*UQCR* genes), and Complex IV (*COX* genes); (**C**) Schematic: Model of aberrant heme metabolism in liver tumors. Heme biosynthesis gene expression is reduced, while protoporphyrin IX (PPIX) accumulation is enhanced, as inferred from an imbalance in heme production (*ALAS1* downregulated, *HMBS* upregulated). Increased *FLVCR1* expression suggests elevated heme/porphyrin export. Cytochrome genes are decreased, in contrast to electron transport chain (ETC)-related genes, which are upregulated in more aggressive cancer cell populations.

High-resolution spatial profiling of liver tumors now provides direct gene expression evidence of both porphyrin overdrive and redirected heme metabolism in primary HCC and liver metastases (Figure 3).

In HCC, comparison of more transformed hepatocyte-derived tumor cells (Hep-like tumor) with more differentiated Hep-pericentral cells revealed reduced expression of the rate-limiting [45] heme biosynthesis enzyme encoding gene *ALAS1* (Figure 3A). Cytochrome P450 (CYP) genes were broadly downregulated, whereas ETC components, particularly cytochrome c oxidase (COX) subunits, were relatively upregulated in the aggressive cell population (Supplemental Table S5).

In liver metastasis, comparison between stem-like outer tumor epithelial cells and the more differentiated tumor core revealed a similar remodeling pattern (Figure 3B). Early biosynthetic gene *ALAD* was downregulated, whereas later pathway genes such as *HMBS* were upregulated. Heme degradation gene *HMOX2* and heme/porphyrin exporter *FLVCR1* were elevated. CYP genes were downregulated, in contrast to upregulated ETC genes from Complex I (*NDUF*), Complex III (*UQCR*), and Complex IV (*COX*).

A schematic model (Figure 3C) summarizes this conserved “porphyrin overdrive” phenotype. In both tumor types, reduced expression of early step (not late step) heme biosynthesis genes alongside upregulation of downstream enzymes suggests protoporphyrin IX (PPIX) accumulation. Elevated *FLVCR1* supports enhanced porphyrin export, while coordinated suppression of CYP genes and induction of ETC subunits indicates a metabolic shift toward oxidative phosphorylation. Together, these shifts suggest a reprioritization of heme metabolism during tumorigenesis, moving from complete heme synthesis toward intermediate accumulation, and from P450-mediated processes toward enhanced ETC activity. This reprogramming appears to represent a shared heme metabolic adaptation that supports tumor progression across distinct hepatic tumor contexts.

### 3.4. Immune Cell Aggregates Diverge in Architecture Between HCC and Liver Metastases

**Figure 4:**
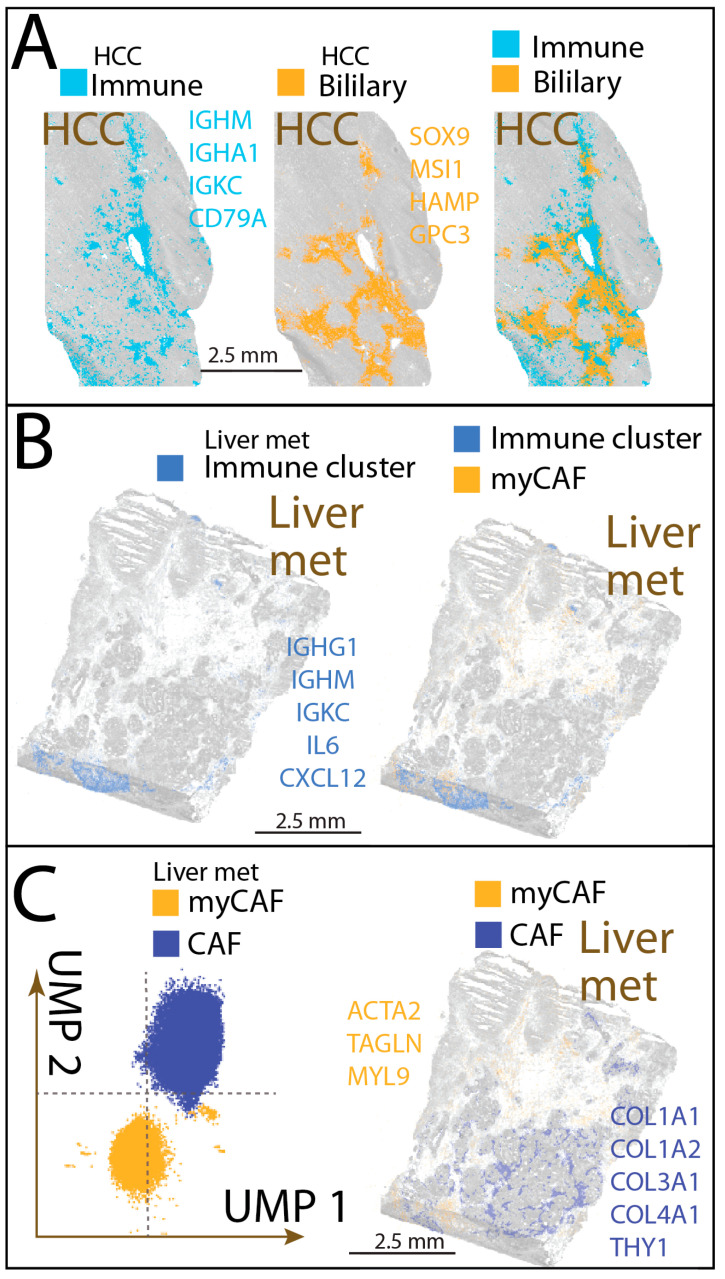
Immune cell aggregates in HCC and liver metastasis. (**A**) In HCC, immune cell populations appear as dispersed clusters along boundaries between different cell type regions, particularly at interfaces with biliary-like cell groups. This cell population harbors B-lineage cells expressing *IGHM*, *IGHA1*, and *CD79A*. Adjacent biliary-like cells express markers such as *SOX9* and *HAMP*; (**B**) In liver metastasis, immune cells form scattered clusters throughout the tissue and are frequently adjacent to myofibroblastic cancer-associated fibroblast (myCAF) populations. These immune clusters show elevated expression of *IL6* and *CXCL12*; (**C**) Two distinct CAF populations are identified in liver metastasis. These CAF types form separate clusters in UMAP space, independent of their physical spatial location. CAFs are predominantly located in the metastatic invasion zone, whereas myCAFs are enriched in the metastatic germline-like dedifferentiation zone. myCAFs express *MYL9* in addition to classic fibroblast genes.

Spatial transcriptomic analysis revealed that immune cell clusters were present in both HCC and liver metastases, but their spatial positioning, stromal associations, and molecular composition were markedly distinct (Figure 4).

In HCC, immune cell clusters appeared as dispersed clusters positioned at the boundaries between distinct epithelial populations, particularly at interfaces with biliary-like cell groups expressing *SOX9* and *HAMP* [46,47]. These immune aggregates showed a prominent B cell component, spanning a continuum from mature B cells to plasma cells, with strong expression of canonical B cell marker genes including *IGHM*, *IGKC*, *IGHG1*, and *IGHA1*. Chemokine genes such as *CCL19*, were co-expressed with fibroblastic genes [48] including *ACTA2* and *COL1A1*, creating a composite niche of B cells, plasma cells, stromal elements, and vascular structures. Histologically, these immune clusters were located near bile ducts and vascular-rich regions, suggesting integration into liver-specific stromal and vascular frameworks and a potentially transient role in immune regulation.

In liver metastases, immune clusters were more broadly distributed and more frequently associated with myofibroblastic cancer-associated fibroblasts (myCAFs) than with vascular structures. At the Zone Invasion’s front, immune clusters were embedded within fibroblastic networks enriched for extracellular matrix remodeling genes [49] such as *LOX*, and *LOXL2*, forming a scaffold reminiscent of fibroblastic reticular cells in secondary lymphoid organs. In Zone Plasticity, immune clusters were occasionally situated adjacent to dedifferentiated tumor niches, co-localizing with myCAFs expressing *MYL9* [50] alongside canonical fibroblast markers. These metastasis-related immune cells expressed IL6 and *CXCL12*, cytokines that promote immune and stromal interactions and support immune cell maturation in settings of chronic inflammation and fibrosis.

Together, these observations indicate that HCC immune clusters are vascular-integrated, perivascular immune–stromal hubs, whereas liver metastasis immune clusters are matrix-embedded within fibrotic scaffolds, with stromal composition and positioning differing between Zone Invasion and Zone Plasticity. These architectural and molecular distinctions suggest divergent mechanisms of immune cell cluster maintenance, potentially influencing their stability, immune surveillance capacity, and responsiveness to immunotherapy.

### 3.5. Germline/Neural-like Plastic Tumor Dedifferentiation Hub in Liver Metastasis

**Figure 5:**
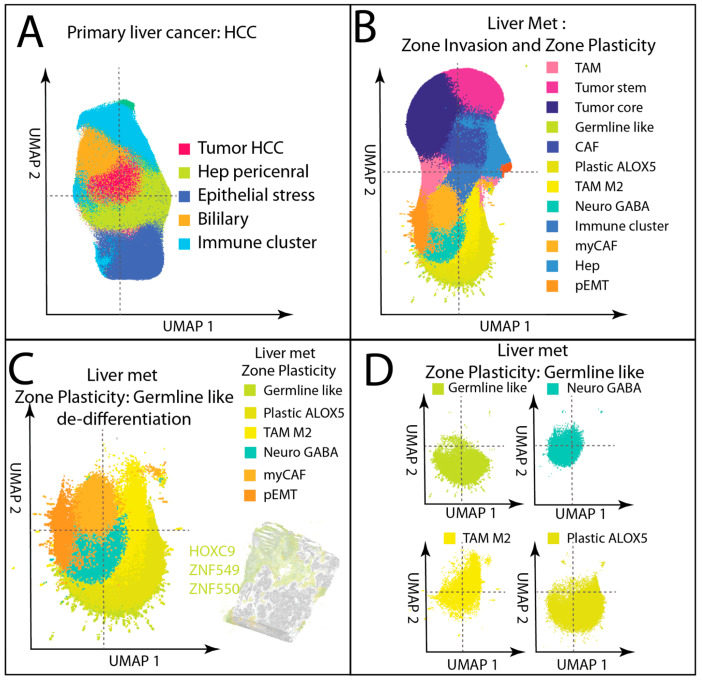
Increased cellular complexity in liver metastasis compared to HCC, driven by highly plastic cells in Zone Plasticity (germline-like dedifferentiation zone). (**A**) UMAP of HCC cell types, generated from gene expression profiles without incorporating spatial coordinates, shows that the majority of cells are differentiated; (**B**) UMAP of liver metastasis reveals markedly more complex cellular lineages; (**C**) UMAP of Zone Plasticity in liver metastasis (germline-like dedifferentiation zone) shows cell types lacking differentiation features and displaying high plasticity. These include germline-like cells, plastic *ALOX5*-positive cells, M2-like TAMs, Neuro GABA cells, myCAFs, and pEMT cells. Germline-like cells express genes such as *HOXC9* and *ZNF* genes; (**D**) UMAP of individual cell types within Zone Plasticity of metastasis, including germline-like cells, Neuro GABA cells, M2-like TAMs, and plastic *ALOX5*-positive cells, show overlapping or adjacent positions, indicating intrinsic relatedness in their lineage or state transitions.

Cell state diversity was markedly greater in liver metastasis than in HCC. In HCC, UMAP of single-cell transcriptomes, derived solely from gene expression without spatial coordinates, revealed a relatively limited number of malignant and stromal states (Figure 5A). By contrast, the same approach in liver metastasis produced a far more intricate arrangement of cell lineages (Figure 5B), reflecting the heterogeneous populations of Zone Invasion and, most strikingly, the emergence of a dedifferentiation niche, Zone Plasticity.

In liver metastasis, this Zone Plasticity “germline-like” hub was a spatially cohesive structure within the germline-like dedifferentiation zone defined in Section 3.2, positioned deep within the tumor mass away from the structured invasive front. High-resolution spatial mapping (Figure 5C) identified a network of poorly differentiated, highly plastic cell states concentrated in this hub. Germline-like tumor cells expressed genes indicative of developmental/germline-associated reprogramming, such as *HOXC9*, *ZNF549*, and *ZNF550* [51,52]. Neuro-GABA tumor–stromal hybrids displayed neuronal signaling components (*GABBR1*) [53,54], suggesting neural mimicry and stress-adaptive signaling within the tumor stroma. M2-like TAMs populated the hub with an immunoregulatory, pro-angiogenic phenotype enriched for scavenger receptors, angiogenic factors, and checkpoint molecules, often positioned alongside plastic tumor states. pEMT-like tumor cells co-expressed epithelial (*KRT20*) and mesenchymal/dedifferentiation related genes (e.g., *ASXL3 and PRM2*, *both among the top 20 upregulated genes*), consistent with partial epithelial-to-mesenchymal transition at invasive or dedifferentiated margins. myCAFs contributed an expression signature of ECM-contractile scaffold (*COL1A1*, *ACTA2*, *MYL9*) frequently integrated with fibroblastic networks embedded in the Zone Plasticity matrix. Plastic-*ALOX5*^+^ tumor cells exhibited an inflammatory, lipid-metabolic program (*ALOX5*) coupled to germline-associated genes (*TDRD10*) [55,56], linking inflammatory lipid metabolism to stem-like tumor plasticity.

UMAP focusing on liver metastasis Zone Plasticity (Figure 5D) revealed substantial overlap and adjacency between these lineages in low-dimensional space, suggesting shared lineage trajectories or dynamic interconversion. Their co-localization in both physical space and transcriptional state space supports the interpretation that Zone Plasticity forms a phenotypic plasticity hub unique to advanced liver metastases. This microenvironment, where tumor, stromal, and immune cells undergo reciprocal reprogramming, may act as a driver of therapeutic resistance and a focal point for metastatic progression.

Notably, no equivalent germline-like plasticity hub was detected in HCC, showing a fundamental architectural and evolutionary difference between primary liver tumors and metastatic tumors in the hepatic environment.

### 3.6. Cross-Validation of Porphyrin- and Signaling Lipid-Related Protein Expression in Human Liver Tissues and Survival Data

**Figure 6:**
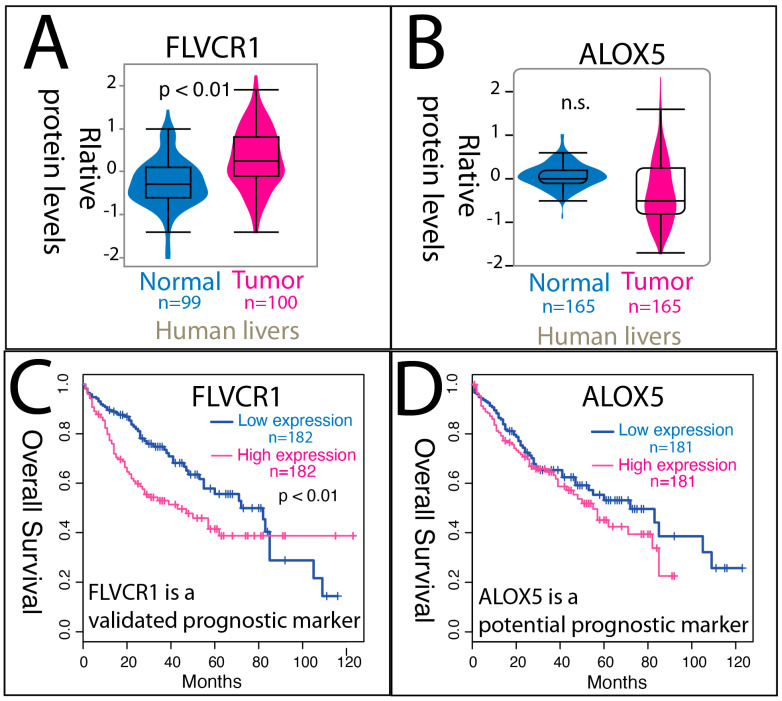
Protein expression and prognostic significance of FLVCR1 and ALOX5 in human liver tumors. (**A**) Protein expression data from 99 normal and 100 tumor liver samples (HMP dataset). (**B**) Protein expression data from 165 normal and 165 tumor liver samples (HMP dataset). FLVCR1 protein levels are significantly different between tumors and normal tissues (nonparametric Kruskal–Wallis test, *p* < 0.01). (**C**) Kaplan–Meier analysis of FLVCR1 overall survival, demonstrating its value as a validated prognostic marker. Results were confirmed in an independent validation set. (**D**) Kaplan–Meier analysis of ALOX5 overall survival, showing that ALOX5 expression is a potential prognostic marker. KM curves were generated from TCGA liver cancer datasets.

To further validate the aberrant heme metabolism and upregulated inflammatory lipid gene expression patterns observed in our high-resolution spatial transcriptomic analysis, we examined public human protein and clinical datasets. Protein expression data from the Human Protein Atlas (HMP v23.0) revealed consistent differences in liver cancer: analysis of 99 normal and 100 tumor samples, as well as 165 normal and 165 tumor samples, showed significantly elevated FLVCR1 protein levels in tumors compared with normal tissues (nonparametric Kruskal–Wallis test, *p* < 0.01) (Figure 6A,B). ALOX5 protein expression was also elevated in tumors, though it did not reach statistical significance.

To assess clinical relevance, Kaplan–Meier survival analyses were performed (Figure 6C,D). *FLVCR1* expression was significantly associated with overall survival in TCGA liver cancer datasets (GDC, v43.0) and this association was independently validated in a survival dataset listed in HMP, supporting its designation as a *validated prognostic marker*. In contrast, *ALOX5* expression showed a significant association with survival in TCGA liver cancer datasets but has not yet been replicated in an independent dataset, and thus is considered a *potential prognostic marker*. Together, these analyses provide orthogonal validation of our transcriptomic findings and highlight *FLVCR1* and *ALOX5* as clinically relevant candidates for further investigation.

### 3.7. Distant Liver Metabolic Reprogramming Gene Expression Mirrors Tumor-Localized Processes in Murine Models

Our high-resolution spatial profiling of primary HCC and liver metastases suggested a conserved *porphyrin overdrive* phenotype, characterized by altered heme metabolism, increased porphyrin export, and inflammatory lipid signaling (Section 3.3). To determine whether these changes were confined to tumor tissue or could also occur in tumor-free liver, we analyzed an independent previously generated single-cell dataset from tumor-bearing mice in which the liver was not a direct site of tumor growth [31].

**Figure 7:**
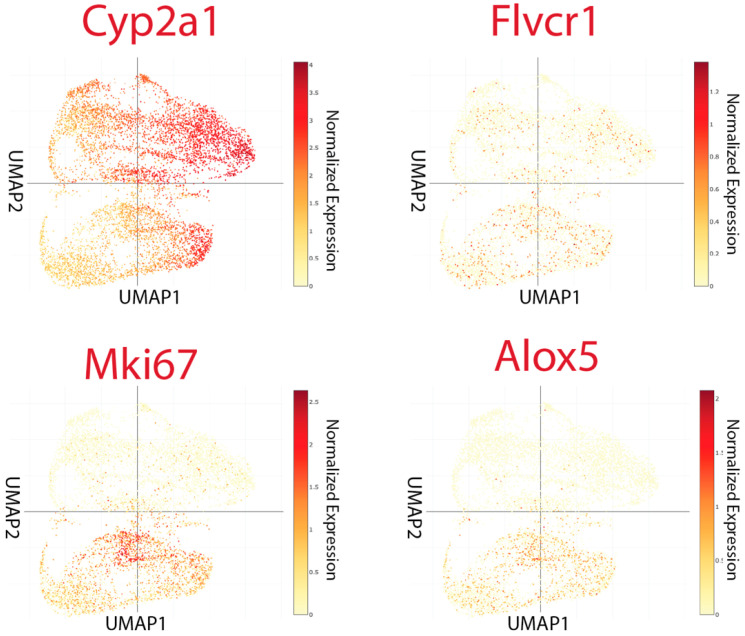
Tumor-bearing mice exhibit distant liver metabolic reprogramming in heme and prostaglandin pathways. UMAP of liver cell populations from two normal mice (upper cluster) and two tumor-bearing mice (lower cluster), reanalyzed from Vandenbon et al. In both groups, *Cyp2a1* expression was maintained (fold change < 2), reflecting conserved hepatic metabolic activity. In contrast, livers from tumor-bearing mice exhibited significant upregulation of *Mki67* (adjusted *p* < 0.05), indicating enhanced cell cycle activity. Moreover, the heme/porphyrin export gene *Flvcr1* (adjusted *p* < 0.05) and the prostaglandin pathway enzyme *Alox5* (adjusted *p* < 0.05) were elevated in tumor-bearing livers, consistent with metabolic reprogramming. These changes suggest that tumors can trigger early (“pioneer”) signals of heme and lipid pathway activation in distantly affected organs.

UMAP of liver cell populations from two normal mice and two tumor-bearing mice showed a clear segregation between the two groups, with normal liver cells forming an upper cluster and tumor-bearing liver cells occupying a distinct lower cluster (Figure 7). In both conditions, *Cyp2a1* expression was retained (fold change < 2), reflecting conserved baseline hepatic metabolic activity. However, livers from tumor-bearing mice exhibited marked upregulation of *Mki67*, indicating increased proliferative activity [57].

Strikingly, two hallmarks of the tumor-localized metabolic signature, upregulation of the heme/porphyrin export transporter *FLVCR1* and the prostaglandin pathway enzyme *ALOX5*, were also elevated (adjusted *p* < 0.05, fold change > 2) in the tumor-free livers of tumor-bearing mice. This convergence suggests that the metabolic rewiring seen in aggressive hepatic tumor cells can also be systemically induced in distant liver tissue.

These findings imply that the presence of a tumor at a remote site is sufficient to trigger early (“pioneer”) metabolic adaptations in the liver, potentially priming the organ for systemic inflammatory or metabolic stress responses. Such distant activation of porphyrin export and inflammatory lipid metabolism may represent an underappreciated component of host–tumor cross-talk and could influence the establishment of metastatic niches or the systemic metabolic state that supports tumor progression.

## 4. Discussion

Our study presents a high-definition spatial transcriptomic comparison of primary HCC and liver metastases in an exploratory study, revealing both conserved metabolic adaptations and tumor-type-specific architectural features. The results were cross-compared with an independently generated mouse dataset. Our findings align with and extend recently published spatial transcriptomic studies of liver metastases [58,59], which, although performed at lower resolution and with differing focal points, consistently reported epithelial invasion zones and metabolic heterogeneity. Together, these comparisons situate our high-definition dataset within the broader landscape of emerging liver cancer spatial atlases and highlight both shared features and the unique resolution gained from Visium HD profiling.

A key finding is the contrasting architecture and lineage dispersion between HCC and liver metastases. We observed that, in HCC, more differentiated Hep-pericentral cells confined to a localized region and more transformed Hep-like cells dispersed in discrete foci. This suggests that transformation level may be linked to physical spread within the tumor parenchyma. In contrast, we found that liver metastases exhibited a sharply compartmentalized architecture with a structured invasive front (Zone Invasion) and a deep-seated dedifferentiation niche (Zone Plasticity). The definition of these zones, anchored by distinct tumor, immune, and stromal populations, supporting heterogeneity reported before [58,60,61,62,63,64,65], provides a new framework for interpreting metastatic microenvironments in the liver.

Another distinctive observation is the germline/neural-like plasticity hub in liver metastases. Such a niche was absent in HCC. This suggests that the formation of such a plasticity hub may be a metastatic adaptation of invading tumors to the hepatic environment. Our findings align with the idea that metastatic tumors can generate organ-specific “secondary niches,” distinct from those in primary sites, to facilitate long-term survival and progression [66,67,68,69,70,71].

We propose a *Checkpoint Intermediate Model* of metabolic stress signaling, in which unstable metabolites such as heme intermediates and ceramide intermediates, which can act as toxic accumulants, and PUFA-derived intermediates, which primarily function as signaling mediators, accumulate at downstream bottlenecks and serve as checkpoint signals. Support for this model comes from our analysis of an independent murine dataset in Section 3, which showed distant liver metabolic reprogramming in tumor-bearing mice, with *FLVCR1* and *ALOX5* upregulated in tumor-free liver. These findings supports the concept that tumors can systemically prime distant sites for colonization [72]. In our model, unstable intermediates broadcast metabolic stress both locally and systemically, linking core metabolic flux to inflammatory and survival pathways.

This model can be understood in a proposed *spark*–*carrier*–*echo* framework. Porphyrins and PUFA mediators act as short-lived “*sparks*”, normally neutralized or degraded within minutes [15,73]. When tumors overwhelm scavenging or catabolic systems, these sparks may be stabilized in circulation or converted into secondary “*carriers*” such as cytokines [74]. The “*echoes*” are the downstream systemic responses, fever, acute-phase proteins, immune suppression, or proliferative reprogramming [75,76] that extend tumor influence beyond the local niche.

While our analyses highlight a potential conserved reprogramming of heme and lipid pathways, we recognize that direct measurement of the corresponding signaling metabolites remains challenging. Many porphyrin intermediates and PUFA-derived lipid mediators are chemically unstable, exist at concentrations orders of magnitude lower than structural metabolites [73,77,78]. As such, they are rarely detected in untargeted metabolomics datasets and often require specialized targeted assays to quantify reliably. Consequently, transcriptomic evidence provides a feasible first step to identify candidate pathways, but future studies integrating targeted metabolomics and functional assays will be essential to confirm the presence and dynamics of these labile signaling molecules.

Future work should build on these insights by determining whether the conserved heme–lipid metabolic adaptations shared by both primary and metastatic tumors can be therapeutically targeted, and by testing whether systemic metabolic reprogramming in histologically normal liver can serve as an early biomarker or modifiable node for preventing metastatic spread. Our findings highlight *FLVCR1* and *ALOX5* as spatially organized and clinically relevant genes in liver cancer, but their therapeutic potential remains exploratory. *FLVCR1*, a heme exporter, has been shown in other cancer contexts to promote survival under oxidative stress, with knockdown reducing proliferation and inducing apoptosis [79,80]. ALOX5, a lipoxygenase in leukotriene biosynthesis, has been linked to tumor growth and angiogenesis, and pharmacological inhibition with Zileuton has shown antitumor effects in preclinical models [81,82]. While these studies suggest that perturbing heme export or lipid signaling could influence tumor behavior, further functional validation in liver cancer is needed before therapeutic implications can be firmly drawn. These findings should be regarded as hypothesis-generating given the limited sample size and absence of direct functional validation, underscoring the need for confirmation in larger human cohorts. Extending these spatial approaches to larger cohorts and integrating with orthogonal methods such as spatial proteomics, multiplex imaging, and metabolomics will be critical to validate the generality of these findings and to translate them into clinical applications.

## 5. Conclusions

High-definition spatial transcriptomics of primary HCC and liver metastases in a two-specimen exploratory study revealed tumor-type-specific architectures alongside potential shared metabolic programs. HCC displayed ordered lineage organization with localized transformation gradients, whereas liver metastases formed invasive and dedifferentiation niches, including a germline/neural-like spatial plasticity hub absent in HCC. Both tumor types exhibited a candidate “porphyrin overdrive” program, characterized by altered gene expressions related to heme metabolism and inflammatory lipid production.

These transcriptomic observations are supported by orthogonal evidence: public human proteomic data demonstrate differential FLVCR1 and ALOX5 expression in liver cancer, and survival analyses in large patient cohorts associate these genes with overall survival. While these findings highlight potential conserved vulnerabilities, they remain hypothesis-generating given the limited cohort size. Prior studies suggest that perturbation of *FLVCR1* and *ALOX5* can influence tumor growth and survival in other cancer contexts, raising the possibility of therapeutic relevance, although this remains speculative without functional validation in liver cancer. Together, these spatially resolved cellular and metabolic programs, supported by proteomic and survival evidence, provide a framework for future investigations and may ultimately inform strategies to target both local and systemic tumor–host interactions.

## 6. Limitations

The strength of this work lies in presenting the first side-by-side, high-definition spatial transcriptomic comparison of primary HCC and liver metastasis, analyzed at the highest resolution currently available. This allowed us to map rare cellular states and define fine-scale architectural features that have not been resolved in prior studies.

That said, several limitations should be noted. First, the analysis is based on a pilot study of one primary tumor and one metastasis, which provides depth of resolution but limits generalizability. The structures, such as the liver metastasis plasticity hub and the conserved porphyrin overdrive program, require validation in larger and more diverse patient cohorts. Second, the samples were archival, likely reflecting late-stage tumors with extensive growth; thus, the features described here may over-represent advanced disease, and the absolute tissue-of-origin of the metastasis cannot be fully resolved. Third, while we provide orthogonal support from public human proteomic and survival data, the current study relies primarily on transcriptomic analysis at the bin level rather than true single-cell resolution. Functional validation at the protein or metabolite level, for example, through spatial proteomics, metabolomics, or targeted assays, will be essential in future work. Finally, the murine re-analysis provides preliminary insights into potential pioneer metabolic signals, but these observations remain unvalidated in human tissues.

Despite these caveats, the study establishes a blueprint for dissecting tumor ecosystems with high spatial resolution and serves as a foundation for mechanistic validation and translational applications.

## Data Availability

Data needed to evaluate the conclusions in the paper are available in the Supplementary Materials and in NCBI SRA.

## Supplementary Materials

The following supporting information can be downloaded at: https://www.mdpi.com/article/10.3390/cancers17193210/s1. Supplemental Tables S1–S5 are available.
